# Red meat consumption and risk factors for type 2 diabetes: a systematic review and meta-analysis of randomized controlled trials

**DOI:** 10.1038/s41430-022-01150-1

**Published:** 2022-05-05

**Authors:** Lisa M. Sanders, Meredith L. Wilcox, Kevin C. Maki

**Affiliations:** 1 Midwest Biomedical Research, Addison, IL 60101 USA; 2 Indiana University, Department of Applied Health Science, School of Public Health, Bloomington, IN 47401 USA

**Keywords:** Type 2 diabetes, Biochemistry

## Abstract

**Background and objectives:**

Results from observational studies suggest an association of red meat intake with risk of type 2 diabetes mellitus (T2D). However, results from randomized controlled trials (RCTs) have not clearly supported a mechanistic link between red meat intake and T2D risk factors. Therefore, a systematic review and meta-analysis were conducted on RCTs evaluating the effects of diets containing red meat (beef, pork, lamb, etc.), compared to diets with lower or no red meat, on markers of glucose homeostasis in adults.

**Methods:**

A search of PubMed and CENTRAL yielded 21 relevant RCTs. Pooled estimates were expressed as standardized mean differences (SMDs) between the red meat intervention and the comparator intervention with less or no red meat.

**Results:**

Compared to diets with reduced or no red meat intake, there was no significant impact of red meat intake on insulin sensitivity (SMD: −0.11; 95% CI: −0.39, 0.16), insulin resistance (SMD: 0.11; 95% CI: −0.24, 0.45), fasting glucose (SMD: 0.13; 95% CI: −0.04, 0.29), fasting insulin (SMD: 0.08; 95% CI: −0.16, 0.32), glycated hemoglobin (HbA1c; SMD: 0.10; 95% CI: −0.37, 0.58), pancreatic beta-cell function (SMD: −0.13; 95% CI: −0.37, 0.10), or glucagon-like peptide-1 (GLP-1; SMD: 0.10; 95% CI: −0.37, 0.58). Red meat intake modestly reduced postprandial glucose (SMD: −0.44; 95% CI: −0.67, −0.22; *P* < 0.001) compared to meals with reduced or no red meat intake. The quality of evidence was low to moderate for all outcomes.

**Conclusions:**

The results of this meta-analysis suggest red meat intake does not impact most glycemic and insulinemic risk factors for T2D. Further investigations are needed on other markers of glucose homeostasis to better understand whether a causal relationship exists between red meat intake and risk of T2D.

**PROSPERO registration:**

CRD42020176059

## Introduction

Results from observational studies suggest that dietary patterns higher in red meat intake, compared to patterns lower in red meat, are associated with increased risk for T2D [1–4]. However, results from RCTs have been mixed in demonstrating a mechanistic link between red meat intake and the T2D risk factor profile [5–7]. Furthermore, the recommendations for red meat intake have differed among scientific organizations. The 2020 Dietary Guidelines Advisory Committee report reconfirmed the findings of the 2015 Dietary Guidelines Advisory Committee that there is “moderate” scientific evidence indicating a dietary pattern limiting intake of red and processed meat, sugar-sweetened foods/drinks, refined grains and high-fat dairy and higher in fruits, vegetables, and whole grains reduces the risk of T2D [8]. However, the Nutritional Recommendations (NutriRECS) Consortium evaluated 23 cohort studies with 1.4 million participants and concluded there was low to very-low certainty of evidence that decreasing unprocessed red meat or processed meat intake may result in very small reductions in the risk for type 2 diabetes [6, 9].

Insulin resistance is one of the characteristics of T2D and several controlled feeding studies have evaluated the impact of higher protein or red meat intakes on whole-body insulin sensitivity, compared to diets with lower red meat intake [10–14]. While insulin sensitivity is one key determinant of glucose tolerance, others include pancreatic beta- and alpha-cell function, hepatic glucose production, adipose tissue lipolysis, and incretin responses to food intake [10, 15–18]. Relatively little research has been completed in humans to assess possible mechanistic links between red meat intake and determinants of glucose tolerance other than insulin sensitivity. The purpose of this review and meta-analysis is to assess the status of the available data on mechanisms through which red meat intake might affect T2D risk from dietary intervention studies in humans, with a focus on the established determinants of glucose tolerance [10].

## Materials and methods

### Literature searches

The Preferred Reporting Items for Systematic Reviews and Meta-Analyses (PRISMA) guidelines were followed for performing the systematic review and meta-analyses [19]. A comprehensive literature search was conducted using the PubMed and CENTRAL databases to identify papers through December of 2021. The search was designed to identify publications of RCTs that examined red meat intake and outcomes related to risk factors for T2D, including insulin sensitivity [e.g., Matsuda Index, Quantitative Insulin Sensitivity Check Index (QUICKI), Predicted M (PREDIM), homeostatic model of insulin sensitivity (HOMA-2%S), insulin resistance [e.g., homeostatic model of insulin resistance (HOMA-IR)], fasting glucose and insulin, postprandial glucose and insulin, pancreatic beta-cell function, markers of lipolysis (e.g. free fatty acids), incretins (e.g., GLP-1, gastric inhibitory peptide), insulin-like growth factor-1, serine kinases, hepatic glucose production and uptake, glucagon, kidney glucose reabsorption, branched-chain amino acids and trimethylamine N-oxide. Full search term details are provided in Supplemental Table 1.

### Inclusion and exclusion criteria

Inclusion criteria consisted of RCTs conducted in adult humans (≥18 y of age), English language publications, fresh/minimally processed or further processed red meat (e.g., beef, pork, lamb, etc.) as the main intervention compared to a control without red meat or with a lower level of red meat, and a baseline and post-treatment measure of fasting and/or postprandial glycemia, insulinemia, insulin sensitivity, pancreatic beta-cell function, or other determinants of glucose tolerance such as hepatic glucose output, incretin responses, or free fatty acid levels in circulation. Exclusion criteria included observational studies (cross-sectional, retrospective, or prospective cohorts), case-control or single-arm studies with no control condition, studies in animals or in vitro, multicomponent interventions where the effect of red meat cannot be determined (e.g., energy-restricted diets), studies comparing different types of red meat without a lower or no red meat control diet, studies based on red meat components (e.g., iron, carnosine) or dietary supplements, interventions administered via tube feeding or enteral nutrition, studies in children (<18 y of age) or pregnant/lactating women, and studies in subjects with a chronic disease, with the exception of T2D, overweight/obesity, prediabetes, or metabolic syndrome. These chronic diseases were included as they are related to increased risk of T2D. The original protocol excluded interventions with mixed animal proteins compared to diets lower in animal proteins; however, this led to the exclusion of several studies that varied red meat intake. Therefore, the protocol was altered to include studies comparing higher and lower animal protein intake and to include a subgroup analysis on studies varying only in red meat intake.

### Screening and data extraction

After screening of titles and abstracts, full texts of all publications identified as potentially eligible were obtained for further review by two independent reviewers. Publications that were unclear with respect to eligibility were resolved by discussion with the research team. Reference lists from eligible publications were reviewed to determine any additional studies for inclusion. Following the full-text review, PICO (population, intervention, comparator, and outcome) data were extracted from the eligible studies into a database by one reviewer and verified for accuracy by a second reviewer. All discrepancies were resolved by discussion among the reviewers and referencing the original publication. In studies where outcomes were reported in bar graphs, Engauge Digitizer software version 4.1 (http://markummitchell.github.io/engauge-digitizer/) was used to estimate the means and standard deviation (SD) or standard error of the mean (SEM) in the graphs for inclusion in the database. If studies did not report SDs or SEMs for an outcome, the SD was estimated as the maximum SD reported by other studies with the same outcome. Sensitivity analyses were completed to assess different SD assumptions for such studies. Since results did not materially differ, only those with maximum SD are presented.

### Assessment of study quality

Risk of bias for each relevant outcome within a study was assessed with the Cochrane risk of bias tool for randomized trials [20]. The quality of the evidence for each outcome was assessed using the Grading of Recommendations Assessment, Development and Evaluation (GRADE) method [21].

### Statistical analysis

Meta-analyses were completed using Comprehensive Meta-analysis (CMA) Software version 3 (Biostat, Englewood, NJ). Insulin sensitivity was pre-specified as the primary outcome. All insulin sensitivity measures were analyzed together (Matsuda Index, QUICKI, PREDIM, HOMA-2%S, insulin sensitivity index, and inverse of HOMA-IR) as were all beta-cell function measures (HOMA-2%B, Disposition Index). HOMA-IR was also analyzed separately as a marker of insulin resistance. The primary analysis used pooled SMD estimates (red meat diet vs. lower or no red meat diet) and 95% confidence intervals (CI) for insulin sensitivity, HOMA-IR, fasting glucose and insulin, glycated hemoglobin (HbA1c), pancreatic beta-cell function, and AUC of postprandial glucose, insulin, and GLP-1. Statistical significance for individual study and pooled SMDs was declared when the 95% CI did not include the null value of 0 (i.e., *p* value < 0.05). SMDs express the effect size relative to the standard deviation and do not have units. This approach enables pooling of measurements taken using different scales and measurement techniques (e.g., Matsuda index, QUICKI, PREDIM, etc.) [22]. SMDs should not be interpreted as true differences in means between the test and control because the difference in means is expressed as a fraction of a standard deviation. Studies were weighted according to the inverse of the variance of each study’s effect using random effects models. Random effects models were chosen due to the heterogeneity in study designs, populations, dietary interventions, and study length. Sensitivity analyses included fixed effects models and removal of one study at a time to determine if any study was substantially influencing the results. Subgroup analyses were performed for the type of animal protein in the intervention (red meat only or mixed animal protein), amount of red meat consumed (≤ median or > median of the included studies), comparator diet (plant protein, refined carbohydrate, other animal protein), and health status (healthy, overweight/obese, metabolic dysfunction, T2D). Metabolic dysfunction was defined as subjects with metabolic syndrome or at least one indicator of impaired cardiometabolic health, including impaired glucose tolerance, impaired fasting glucose, or hypertension. The magnitude of effect sizes was interpreted as <0.40 = small, 0.40–0.70 = moderate and >0.70 = large [23].

Data from parallel and crossover RCTs were entered into the CMA software using the following data entry formats: “Independent groups (means, SD)” and “Paired groups (means, SD),” respectively. SMDs and corresponding SEMs for individual studies were computed by the software using the methods for independent groups and matched groups described by Borenstein et al. [24] and an imputed between-treatment correlation of 0.50 for matched groups. Sensitivity analyses were performed for the primary analyses assuming no between-treatment correlation for matched groups and the results were similar. For multiple comparisons within a study (i.e., comparisons that shared a common active or control), individual effect size estimates and variances were computed for all comparisons within a study. A pooled effect size estimate was then computed as the weighted average of the individual effect size estimates. The corresponding pooled effect variance was computed as the variance of a linear combination of two or more variables (i.e., average of two or more effect size estimates) using between-comparison correlations equivalent to the weighted average of the between-active (red meat) correlation and the between-control correlation [25]. A single pooled effect size estimate and variance was then included in the analysis for each study with multiple comparisons. When multiple comparisons included in a subgroup varied from those included in the primary analysis, the above steps were repeated prior to running the subgroup analysis. In the analysis of insulin sensitivity, individual effect size estimates and variances were pooled similarly for studies that reported multiple measures of insulin sensitivity.

Statistical heterogeneity across studies was assessed using Cochran’s *Q* and the *I*^2^ statistic. An *I*^2^ value of ≥40% was used to designate moderate or higher heterogeneity, in accordance with the recommendations in the Cochrane Handbook [26]. Chi-square test statistics and *p* values are reported for Cochran’s *Q* tests for heterogeneity across studies and between subgroups. The presence of publication bias was assessed by Egger’s test and examining funnel plots measuring the SEM as a function of the SMD.

## Results

**Fig. 1:**
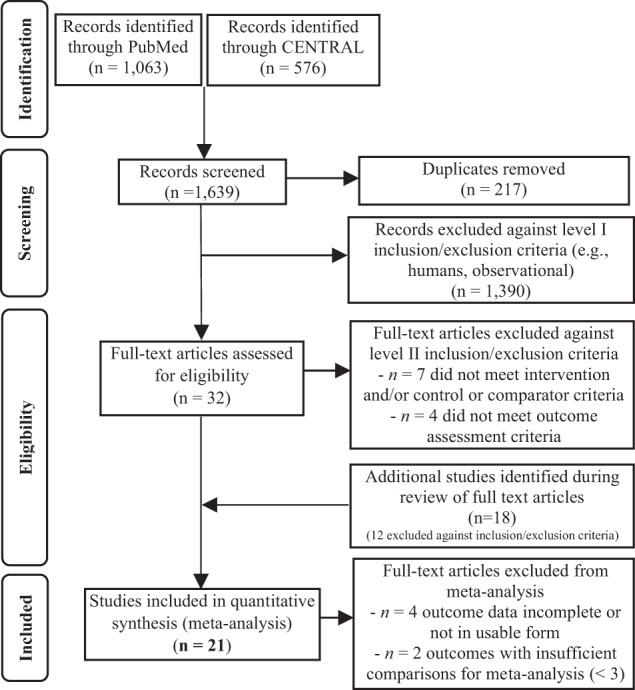
Preferred Reporting Items for Systematic Reviews and Meta-Analyses (PRISMA) flow chart. Flow diagram of the study selection procedure.

A flow diagram summarizing the literature search process is shown in Fig. 1. Thirty-two articles were uncovered in the search and an additional 18 were identified in the reference lists of full-text articles. Twenty-three of these were excluded for not meeting the intervention or comparator criteria or not including an outcome of interest (Supplemental Table 2), leaving 27 articles for inclusion in the systematic review. Of these, 4 included incomplete data (e.g., missing baseline or post-treatment values) or data in an unusable form (e.g., timepoint data vs AUC). Two studies reported additional outcomes of interest, but two comparisons were insufficient to conduct a meta-analysis; therefore, these were excluded (Supplemental Table 2). Overall, 21 studies could be included in the meta-analysis [11–13, 27–44]. The only outcomes with sufficient comparisons (≥3) to include in a meta-analysis were fasting glucose, fasting insulin, insulin sensitivity (all measures), HOMA-IR, postprandial glucose, postprandial insulin, HbA1c, pancreatic beta-cell function, and GLP-1. There were insufficient comparisons (<3) for markers of lipolysis, insulin-like growth factor-1, serine kinases, hepatic glucose production and uptake, glucagon, kidney glucose reabsorption, branched-chain amino acids, and trimethylamine N-oxide.

Study characteristics are in Supplemental Table 3. Fourteen of the studies were crossover [11, 12, 28, 31–37, 39–42] and seven were parallel [14, 27, 29, 34, 38, 43, 44]. Four of the studies were acute (postprandial) feeding trials and the remainder ranged from 1 week [27] to 10 weeks [29], with most studies in the range of 4–8 weeks [11–13, 28, 30, 34, 36, 38–44]. Only seven studies included healthy individuals [27–29, 32, 36, 37, 41], with the remaining studies in subjects with metabolic syndrome, prediabetes, T2D, and/or overweight/obesity. The studies in healthy individuals often included normal weight and overweight or obese subjects but did not stratify the results by BMI classification. The comparator diet to the red meat exposure was different in several studies. Ten studies compared a red meat or mixed animal protein diet to a plant protein diet [14, 27, 28, 31, 32, 34, 37–39, 42], while seven studies compared red meat to other animal protein sources (e.g., dairy, seafood, white meat) [12, 13, 35, 36, 40, 41, 43]. Four studies used red meat to replace refined carbohydrate [11, 29, 33, 44].

### Diets containing red meat and insulin sensitivity

**Fig. 2:**
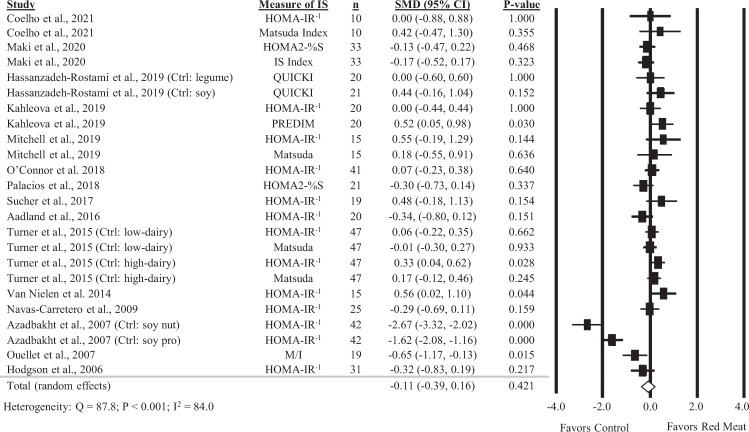
Forest plot of the meta-analysis on the effect of red meat on insulin sensitivity. Values are the standardized mean differences (SMD) for insulin sensitivity measures between diets with red meat intake and diets with less or no red meat intake [11–13, 27, 29–31, 33, 34, 36, 39, 41–44].

Overall, 24 comparisons reported in 15 studies were included in the analysis of the impact of diets containing red meat on insulin sensitivity. There was no significant impact of diets containing red meat, compared to diets with less or no red meat intake, on insulin sensitivity (Fig. 2, SMD: −0.11, 95% CI: −0.39, 0.16) but there was substantial heterogeneity between studies (*Q* = 87.8, *P* < 0.001, *I*^2^ = 84.0%). A sensitivity analysis with one study removed at a time showed no study substantially influenced the results (Supplemental Table 4).

Findings from subgroup analyses are shown in Table 1. There was a small and marginally significant improvement in insulin sensitivity with red meat intake in individuals with T2D (SMD: 0.29; 95% CI: 0.00, 0.57; *P* = 0.049) compared to those consuming less red meat. This was not observed in healthy individuals or individuals with metabolic dysfunction. No other significant differences were found with subgroups evaluating type of meat, amount of red meat intake, or comparator diet.

**Table 1:** Subgroup analysis for the effect of diets containing red meat, compared to less or no red meat intake, on markers of glycemia and insulinemia.

Outcome and subgroups	Comparisons/studies	Effect estimate SMD^a^	95% CI	*I*^2^ (%)	*P* value^a^
*Insulin sensitivity*					
Type of meat					
Red meat alone	16/9	−0.23	−0.60, 0.14	88.8	0.226
All animal proteins	8/6	0.08	−0.35, 0.50	68.0	0.719
Between subgroup heterogeneity (*Q* = 1.1, *P* = 0.286)					
Comparator diet					
Plant protein	10/6	−0.07	−0.93, 0.79	93.2	0.868
Animal protein	8/5	−0.09	−0.34, 0.16	57.8	0.458
Refined carbohydrate	6/4	−0.15	−0.38, 0.07	10.7	0.181
Between subgroup heterogeneity (*Q* = 0.14, *P* = 0.934)					
Health status					
Healthy	6/4	−0.02	−0.31, 0.27	15.6	0.886
Metabolic dysfunction	11/6	−0.54	−1.11, 0.02	92.3	0.057
Type 2 diabetes	5/3	0.29	0.00, 0.57	0.0	**0.049**
Between subgroup heterogeneity (*Q* = 7.8, *P* = 0.050)					
Amount of red meat (median split)					
≤110 g	11/7	−0.26	−0.83, 0.31	91.2	0.366
>110 g	9/5	0.01	−0.23, 0.24	51.3	0.956
Between subgroup heterogeneity (*Q* = 0.7, *P* = 0.392)					
*HOMA-IR*					
Type of meat					
Red meat alone	8/6	0.29	−0.22, 0.81	91.3	0.264
All animal proteins	5/5	−0.12	−0.40, 0.16	7.9	0.420
Between subgroup heterogeneity (*Q* = 1.9, *P* = 0.172)					
Comparator diet					
Plant protein	6/5	0.23	−0.63, 1.10	92.0	0.601
Animal protein	5/4	−0.04	−0.22, 0.13	7.5	0.635
Between subgroup heterogeneity (*Q* = 0.37, *P* = 0.832)					
Health status					
Healthy	4/4	0.07	−0.19, 0.34	0.0	0.580
Metabolic dysfunction	5/3	0.60	−0.57, 1.77	96.1	0.312
Between subgroup heterogeneity (*Q* = 2.7, *P* = 0.448)					
Amount of red meat (median split)					
≤110 g	6/5	0.29	−0.42, 1.01	92.3	0.421
>110 g	5/4	−0.04	−0.25, 0.17	15.1	0.712
Between subgroup heterogeneity (*Q* = 0.77, *P* = 0.381)					
*Fasting glucose*					
Type of meat					
Red meat alone	17/10	0.20	0.00, 0.39	62.3	**0.049**
All animal proteins	6/6	−0.04	−0.33, 0.25	40.1	0.785
Between subgroup heterogeneity (*Q* = 1.8, *P* = 0.183)					
Comparator diet					
Plant protein	14/8	0.09	−0.21, 0.39	71.0	0.564
Animal protein	5/4	0.00	−0.17, 0.17	0.0	0.998
Refined carbohydrate	4/4	0.38	0.15, 0.62	0.0	**0.001**
Between subgroup heterogeneity (Q = 6.9, ***P*** = **0.031**)					
Health status					
Healthy	5/5	0.05	−0.21, 0.30	26.1	0.720
Metabolic dysfunction	13/7	0.34	0.17, 0.51	34.3	**<0.001**
Type 2 diabetes	3/2	−0.47	−0.90, −0.05	0.0	**0.030**
Between subgroup heterogeneity (Q = 18.7, ***P*** < **0.001**)					
Amount of red meat (median split)					
≤113 g	11/8	0.09	−0.16, 0.35	67.0	0.461
>113 g	10/7	0.17	0.01, 0.34	26.9	**0.043**
Between subgroup heterogeneity (*Q* = 0.25, *P* = 0.620)					
*Fasting insulin*					
Type of meat					
Red meat alone	17/10	0.20	−0.13, 0.53	86.3	0.236
All animal proteins	6/6	−0.12	−0.32, 0.09	0.0	0.274
Between subgroup heterogeneity (*Q* = 2.5, *P* = 0.113)					
Comparator diet					
Plant protein	14/8	0.12	−0.39, 0.63	89.1	0.647
Animal protein	5/4	−0.05	−0.24, 0.15	22.7	0.641
Refined carbohydrate	4/4	0.09	−0.13, 0.32	0.0	0.411
Between subgroup heterogeneity (*Q* = 1.0, *P* = 0.603)					
Health status					
Healthy	5/5	0.01	−0.19, 0.22	0.0	0.917
Metabolic dysfunction	13/7	0.30	−0.16, 0.77	90.3	0.203
Type 2 diabetes	3/2	−0.23	−0.65, 0.19	0.0	0.284
Between subgroup heterogeneity (*Q* = 3.9, *P* = 0.267)					
Amount of red meat (median split)					
≤113 g	11/8	0.27	−0.16, 0.70	87.7	0.215
>113 g	10/7	−0.03	−0.20, 0.15	35.2	0.779
Between subgroup heterogeneity (*Q* = 1.6, *P* = 0.211)					

Values in bold indicate a significant effect of diets containing red meat compared to diets with less or no red meat.

^a^Effect estimates and *p* values from random effects models.

### Diets containing red meat and HOMA-IR

**Fig. 3:**
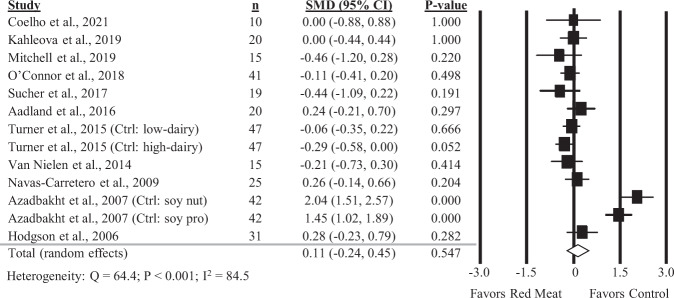
Forest plot of the meta-analysis on the effect of red meat on HOMA-IR. Values are the standardized mean differences (SMD) for HOMA-IR between diets with red meat intake and diets with less or no red meat intake [12, 13, 29, 31, 34, 36, 39, 41, 42, 44].

Overall, 13 comparisons reported in 11 studies were included in the analysis of the impact of diets containing red meat on HOMA-IR. There was no significant impact of diets containing red meat, compared to diets with less or no red meat intake, on HOMA-IR (Fig. 3; SMD: 0.11; 95% CI: −0.24, 0.45) but there was substantial heterogeneity between studies (*Q* = 64.4, *P* < 0.001, *I*^2^ = 84.5%). A sensitivity analysis with one study removed at a time showed no study substantially influenced the results (Supplemental Table 4). No significant differences were found in subgroup analyses (Table 1).

### Diets containing red meat and fasting glucose

**Fig. 4:**
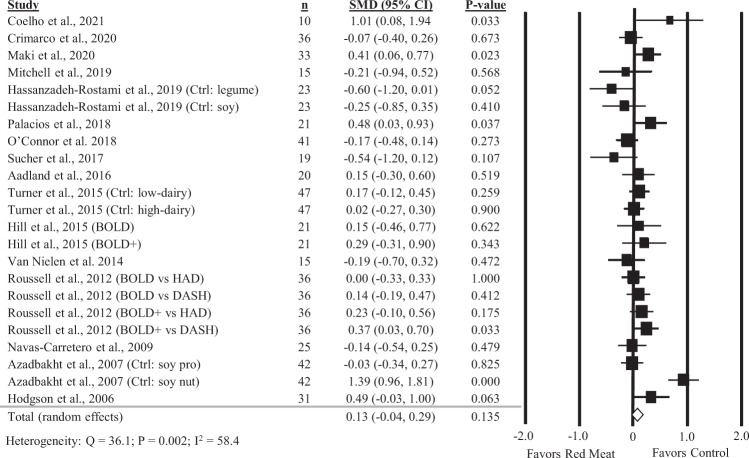
Forest plot of the meta-analysis on the effect of red meat on fasting glucose. Values are the standardized mean differences (SMD) for fasting glucose measures between diets with red meat intake and diets with less or no red meat intake [11–13, 27–30, 33, 34, 36, 38–42, 44].

Twenty-three comparisons reported in 16 studies were included in the analysis of the impact of diets containing red meat on fasting glucose. There was no significant impact of diets containing red meat, compared to diets with less or no red meat intake, on fasting glucose (Fig. 4; SMD: 0.13; 95% CI: −0.04, 0.29) but there was moderate and significant heterogeneity between studies (*Q* = 36.1, *P* = 0.002; *I*^2^ = 58.4%). A sensitivity analysis with one study removed at a time showed no study substantially influenced the results (Supplemental Table 4).

Subgroup analyses showed diets increasing red meat intake alone resulted in a marginally significant increase in fasting glucose (Table 1; SMD: 0.20; 95% CI: 0.00, 0.39; *P* = 0.049) not observed in diets increasing all animal protein intake. Diets containing red meat intake also significantly increased fasting glucose in subjects with metabolic dysfunction (SMD: 0.34; 95% CI: 0.17, 0.51; *P* < 0.001), but the effect size was small. In contrast, diets containing red meat lowered fasting glucose in subjects with T2D (SMD: −0.47; 95% CI: −0.90, −0.05; *P* = 0.030). However, only three comparisons in two studies were included in this subgroup, so these results should be interpreted with caution. Fasting glucose was also significantly increased in studies feeding more than the median intake of red meat (113 g/d) (SMD: 0.17; 95% CI: 0.01, 0.34; *P* = 0.043).

### Diets containing red meat and fasting insulin

**Fig. 5:**
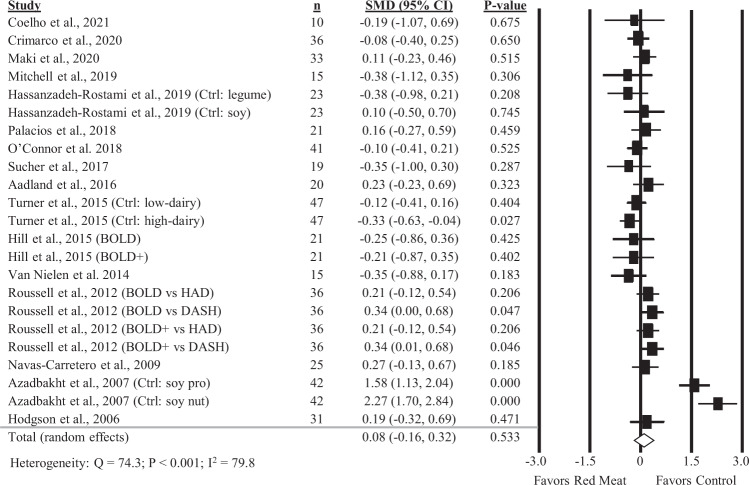
Forest plot of the meta-analysis on the effect of red meat on fasting insulin. Values are the standardized mean differences (SMD) for fasting insulin measures between diets with red meat intake and diets with less or no red meat intake [11–13, 27–30, 33, 34, 36, 38–42, 44].

Twenty-three comparisons reported in 16 studies were included in the analysis of the impact of diets containing red meat on fasting insulin. There was no significant impact of diets containing red meat compared to diets with less or no red meat on fasting insulin (Fig. 5; SMD: 0.08; 95% CI: −0.16, 0.32) but there was substantial heterogeneity between studies (Q = 74.3, *P* < 0.001, *I*^2^ = 79.8%). A sensitivity analysis with one study removed at a time showed no study substantially influenced the results (Supplemental Table 4). No significant differences were found in subgroup analyses (Table 1).

### Diets containing red meat and postprandial glucose and insulin

**Fig. 6:**
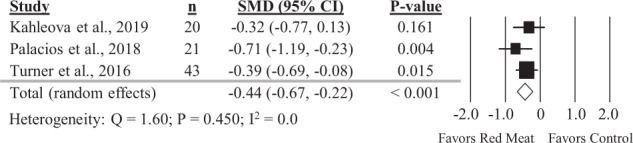
Forest plot of the meta-analysis on the effect of red meat on postprandial glycemic response. Values are the standardized mean differences (SMD) for postprandial glycemic area under the curve (AUC) between diets with red meat intake and diets with less or no red meat intake [31, 33, 35].

Three comparisons reported in three studies were included in the analysis of the impact of diets containing red meat on postprandial glucose. Red meat intake resulted in significantly lower postprandial glucose compared to diets with less or no red meat intake (Fig. 6; SMD: −0.44; 95% CI: −0.67, −0.22; *P* < 0.001) with no significant heterogeneity between studies (*Q* = 1.60, *p* = 0.450, *I*^2^ = 0%). Considering the small number of studies and design differences, caution is warranted in interpretation of these results. For example, one study replaced available carbohydrates with red meat while other studies matched macronutrient content. Also, the populations studied only included subjects with metabolic dysfunction or T2D. A sensitivity analysis with one study removed at a time showed no study substantially influenced the results (Supplemental Table 4). Three comparisons reported in three studies were included in the analysis of the impact of red meat on postprandial insulin. There was no significant effect of red meat intake, compared to diets with less or no red meat, on postprandial insulin (Supplemental Fig. 1; SMD: −0.70, 95% CI: −1.50, 0.11) but with substantial heterogeneity between studies (*Q* = 18.5, *P* < 0.001, *I*^2^ = 89.2%).

### Diets containing red meat and HbA1c, pancreatic beta-cell function, and GLP-1

Few studies included the impact of red meat intake on HbA1c, pancreatic beta-cell function, or GLP-1. There were no significant effects of red meat diets, compared to diets with lower or no red meat on HbA1c (Supplemental Fig. 2), pancreatic beta-cell function (Supplemental Fig. 3), or GLP-1 (Supplemental Fig. 4). There was also no significant heterogeneity between the studies for these outcomes, except for GLP-1 (*Q* = 21.8, *P* < 0.001, *I*^2^ = 86.2%). In a sensitivity analysis, red meat intake significantly reduced GLP-1 with removal of the study by Douglas et al. [37].

### Quality of evidence

The quality of evidence as assessed by GRADE criteria is summarized in Table 2. Overall, the evidence for most outcomes was rated as low to moderate. The evidence rating was downgraded for some outcomes due to concerns about inconsistency, imprecision, and some indirectness. The risk of bias assessments on the individual studies is in Supplemental Table 5. The risk of bias was rated “low” or “some concerns” for most studies. The funding source is not a component in Cochrane ROB; however, a qualitative assessment of results suggested no material differences by funding source category. Ten publications were from studies funded partly or solely by industry or trade associations (animal and plant-based foods) and 11 were funded by the university and governmental sources. Publication bias was not evident for any outcomes, except possibly GLP-1, based on Egger’s regression (Supplemental Table 6).

**Table 2:** Quality of evidence-based on GRADE assessment.

Outcome	Risk of bias	Inconsistency^a^	Indirectness	Imprecision	Publication bias^b^	Decision
Insulin sensitivity	Some concerns	Substantial	Some indirectness	Moderate imprecision	Undetected	⊕⊕_∅∅_ Low
HOMA-IR	Some concerns	Substantial	Some indirectness	Moderate imprecision	Undetected	⊕⊕_∅∅_ Low
Fasting glucose	Some concerns	Substantial	Some indirectness	Moderate imprecision	Undetected	⊕⊕_∅∅_ Low
Fasting insulin	Some concerns	Substantial	Some indirectness	Moderate imprecision	Undetected	⊕⊕_∅∅_ Low
Postprandial glucose	Low	Consistent	No serious indirectness	Moderate imprecision	Undetected	⊕⊕⊕_∅_ Moderate
Postprandial insulin	Low	Substantial	No serious indirectness	Moderate imprecision	Undetected	⊕⊕⊕_∅_ Moderate
HbA1c	Some concerns	Moderate	Some indirectness	Moderate imprecision	Undetected	⊕⊕_∅∅_ Low
Pancreatic beta-cell function	Low	Consistent	No serious indirectness	Moderate imprecision	Undetected	⊕⊕_∅∅_ Low
GLP-1	Low	Substantial	Some indirectness	Moderate imprecision	Possible	⊕_∅∅∅_ Very Low

^a^Based on *I*^2^ using thresholds in Cochrane Handbook for Systematic Reviews of Interventions, Version 6. The Cochrane Collaboration, 2019. Available at: https://training.cochrane.org/handbook/current.

^b^Based on Egger’s regression.

## Discussion

While results from observational studies have suggested an association of red meat intake with T2D incidence, the results of this meta-analysis of RCTs did not show an effect of red meat on glycemic and insulinemic biomarkers associated with the development of T2D. There was no significant effect of diets containing red meat, compared to diets with little or no red meat, on fasting glucose, fasting insulin, postprandial insulin, insulin sensitivity, HOMA-IR, HbA1c, pancreatic beta-cell function, or GLP-1. In a small number of studies, red meat intake lowered postprandial glucose. The lower postprandial glucose may be partly due to the replacement of refined carbohydrate with protein in one study [33], although another study reported higher sugar and lower fiber content in the red meat meal compared to the control meal [31]. With only three studies of varying designs and in subjects with T2D or metabolic dysfunction, more evidence is needed before firm conclusions can be drawn.

Findings from the present meta-analysis are consistent with those reported in a recent review by O’Connor et al. [7] who also found no effect of ≥0.5 servings of red meat/day, compared with <0.5 servings per day, on HOMA-IR, fasting glucose, fasting insulin, and HbA1c in disease-free individuals at risk of T2D. The present meta-analysis included studies in individuals with metabolic syndrome and T2D and additional outcomes of pancreatic beta-cell function and GLP-1, and still did not find an overall effect of red meat intake on markers of glycemia and insulinemia. However, when evaluating outcomes by health status of the participants, some differences in response to red meat intake did emerge. Fasting glucose was significantly increased in individuals with metabolic dysfunction, but the effect was small (SMD 0.34; 95% CI: 0.17, 0.51), corresponding to an increase of approximately 0.12 ± 0.03 mmol/L. The change in fasting glucose did not coincide with changes in insulin sensitivity or resistance. Therefore, the clinical relevance of the increase in fasting glucose in individuals with metabolic dysfunction is uncertain. It is possible that red meat intake may be impacting pancreatic alpha- or beta-cell function or hepatic glucose production, which could increase fasting glucose. Pancreatic beta-cell function was not significantly impacted by red meat intake the present meta-analysis, yet with only three studies and mixed results, the available evidence is limited and more research in this area is needed. Compounds in red meats, such as iron, have been shown to enhance formation of reactive oxygen species, which could plausibly affect pancreatic beta-cell function [45]. Additionally, iron in the liver may impact hepatic insulin extraction, possibly increasing hepatic glucose production [45, 46].

Interestingly, a significant reduction in fasting glucose (SMD = −0.47; 95% CI: −0.90, −0.05) and marginally significant increase in insulin sensitivity (SMD = 0.29; 95% CI: 0.00, 0.57) was observed in subjects with T2D. The decrease in fasting glucose corresponded to 1.02 mmol/L with a SD for baseline fasting glucose of 2.78 mmol/L. However, these results should be interpreted with caution since only 3 studies included subjects with T2D and there were some concerns about quality in two of these studies. One reported a higher baseline fasting glucose and HbA1c in subjects randomized to the animal protein compared to the plant protein diets [14], while another study [34] reported a small but statistically significant decrease in BMI during the interventions, which may have contributed to the trends toward improved fasting glucose reported in these studies. One study report noted that the intervention diets were also healthier than the typical diets consumed, containing approximately 12% more protein and 10% less fat [34]. Thus, there may be effects from providing an overall healthier diet, independent of protein source. These issues underscore the need for high-quality, well-controlled studies in individuals with T2D to better elucidate the effect of red meat intake on glycemia and insulinemia in this population.

The comparator diet, amount of red meat, or type of meat in the intervention (red meat alone or mix of animal proteins) did not generally influence glycemic and insulinemic outcomes. While the overall analysis did not show an impact of red meat on fasting glucose, subgroup analyses demonstrated an increase in fasting glucose in studies increasing red meat alone, studies replacing carbohydrate with red meat, and studies feeding greater than the median intake of red meat (113 g/d). Most studies increasing red meat alone (7 of 10), replacing carbohydrate with red meat (3 of 4), and feeding amounts greater than the median (5 of 7), were in subjects with metabolic dysfunction. In fact, in all subgroups, the only studies reporting directional increases in fasting glucose were in subjects with metabolic dysfunction. Thus, the increase in fasting glucose in these subgroups may be partially explained by the characteristics of the subjects studied (i.e., with metabolic dysfunction but without T2D).

Interestingly, there was no effect on glycemia or insulinemia when red meat was replaced with plant proteins, despite results from epidemiological studies suggesting a positive association between animal protein intake but not plant protein and risk for T2D [47, 48]. Many studies used soy products, which have been associated with a reduced risk of T2D and improvements in glucose homeostasis [49–51]. However, findings from prospective cohort studies have suggested the benefits of soy may be due to isoflavones rather than type of protein [51], and most studies in the current meta-analysis did not evaluate or control the isoflavone contents of the diets.

The results of the current meta-analysis, along with those from other meta-analyses of RCTs [7], are consistent in showing red meat intake does not impact most glycemic and insulinemic risk factors for T2D. However, observational data suggest a positive association between red meat intake and risk for T2D [1, 4, 6, 9, 52, 53]. This discrepancy may partially be explained by other factors associated with red meat intake that may increase the risk for T2D, resulting in residual confounding. Compared to individuals who consume less red meat, those that consume more red meat also tend to be more inactive, eat fewer fruits, vegetables, and whole grains, are more likely to be current smokers, drink alcohol, and have a higher mean BMI [2, 4]. RCTs ensure similar distribution of known and unknown factors associated with the study outcome across groups or conditions and are thus less susceptible to bias and confounding. Most of the studies included in the current meta-analysis also provided unprocessed red meat, which may differentially impact glycemia and insulinemia compared to processed red meat. Many observational studies do not differentiate between unprocessed and processed red meat [1, 9, 53] but those that do tend to find a stronger association of processed meat to risk of T2D [2–4]. Additional studies are needed on processed meats to further elucidate if other processing constituents, such as sodium or nitrates, may be contributing to risk of T2D. It is also possible that mechanisms other than those investigated, or that require exposure over longer periods, could contribute to the observed associations between red meat intake and T2D incidence. Most studies were 4 to 8 weeks in duration so longer studies would be beneficial to understand longer-term impacts of red meat intake. Additional research is needed to investigate effects of red meat consumption on other mechanisms affecting glucose tolerance, particularly pancreatic alpha- and beta-cell function and regulation of hepatic glucose production.

The quality of the evidence for most outcomes was low due to substantial inconsistency, some indirectness, and moderate imprecision. Substantial inconsistency was noted in analyses with high heterogeneity not completely accounted for by subgroup analyses. Some indirectness was identified in that most studies were not in healthy subjects, but rather individuals already at risk for T2D due to other metabolic dysfunctions, including metabolic syndrome or elevated blood lipids. Therefore, to understand if red meat intake influences glycemic and insulinemic outcomes that could increase the risk of developing T2D, it is important to pursue additional studies in healthy individuals without pre-existing conditions or risk factors.

A strength of this meta-analysis is the use of SMDs, which allowed for multiple measurements of insulin sensitivity and insulin resistance to be combined for a more robust analysis. Additionally, multiple subgroup and sensitivity analyses allowed for greater insight into study design factors that may have contributed to the outcomes, including health status, comparator diet, and the amount of red meat in the diet. The exclusion of weight-loss diets may be considered a limitation of the current meta-analysis. However, weight loss can independently impact T2D risk factors, regardless of diet, making it difficult to determine the effects of red meat intake independent of weight loss. The recent meta-analysis by O’Connor et al. [7] included weight loss studies and the findings were similar to those of the present meta-analysis. It is also possible that red meat could be impacting other mechanistic risk factors for T2D, such as hepatic glucose production, or incretins beyond GLP-1, but the paucity of such studies limited the ability to include these outcomes in the analysis. It was not possible to examine the effect of red meat processing and fat content on glycemic and insulinemic outcomes due to most studies including unprocessed red meat or not specifying the type of red meat included in the intervention.

In summary, compared to diets containing less or no red meat, diets containing red meat did not impact glycemic and insulinemic risk factors for T2D, including fasting glucose, fasting insulin, insulin sensitivity, HbA1c, pancreatic beta-cell function, and GLP-1, but did reduce postprandial glycemia in a small number of studies. Individuals with metabolic dysfunction and pre-existing risk factors for T2D may respond differently to red meat intake, as evidenced by an increase in fasting glucose in the subset with metabolic dysfunction and this deserves further investigation. The impact of red meat intake on individuals with T2D should also be further investigated as there were few studies in these individuals, but some indication of improvements in glycemia and insulinemia. These findings reinforce the importance of and need for more high-quality RCTs to provide data relevant to determining whether the association between red meat intake and risk of diabetes is causal.

## Supplementary information

Supplemental Material

## Data Availability

Data reviewed and analyzed during this meta-analysis is available from the corresponding author upon reasonable request.
