# Supplementary material for: Red meat consumption and risk factors for type 2 diabetes: a systematic review and meta-analysis of randomized controlled trials
Source: Eur J Clin Nutr. 2022 May 5;77(2):156–65. doi: 10.1038/s41430-022-01150-1 (PMC9908545; doi:10.1038/s41430-022-01150-1)
Supplement: Supplementary file 1 — Supplemental Material [file 41430_2022_1150_MOESM1_ESM.docx]

**Supplementary Material**

**Red Meat Consumption and Risk Factors for Type 2 Diabetes:**

**A Systematic Review and Meta-Analysis of Randomized Controlled Trials**

LM Sanders, et al.

**Supplemental Tables**

**Supplemental Table 1.** Search Strategy

| **Terms used to identify all studies** | **Additional terms used to limit identified studies** |
| --- | --- |
| **Key search terms for red meat**  “red meat” OR “red meat”[mesh] OR “fresh red meat” OR “processed red meat” OR “unprocessed red meat” OR “meat”[mesh] OR “meat proteins” [mesh] OR “meat products” OR “fresh meat” OR “unprocessed meat” OR “processed meat” OR “minimally processed meat” OR “further processed meat” OR “beef” OR “lamb” OR “mutton” OR “veal” OR “venison” OR “pork” OR “animal protein” OR “animal flesh” OR “poultry”[mesh] OR "poultry" OR "fresh poultry" OR "processed poultry" OR “iron” OR “heme” OR “heme iron”    **Key search terms for T2D mechanistic markers**  “insulin like growth factor-1” OR “IGF-1” OR “incretin” OR “glucagon-like-peptide-1” OR “GLP-1” OR “gastric inhibitory polypeptide” OR “GIP” OR “serine kinase” OR “plasma free fatty acids” OR “FFA” OR “branched chain amino acids” OR “BCAA” OR “leucine” OR “isoleucine” OR “glucose uptake” OR “glucose production” OR “insulin secretion” OR “glucagon” OR “glucose reabsorption” OR “beta cell” OR “pancreas” OR “insulin sensitivity” OR “insulin resistance” OR “hyperglycemia” OR “hyperinsulinemia” AND (“prediabetes” OR “type 2 diabetes” OR “metabolic syndrome”) OR “hepatic glucose production” OR “gluconeogenesis” OR “glucose tolerance” OR “glucoregulation” OR “glucoregulatory” | **Additional filters:** English    randomized controlled trial* OR controlled clinical trial* OR RCT OR random OR placebo* OR control* OR clinical trial* OR clinical research stud* OR random allocation OR double-blind method OR single-blind method OR comparative stud* OR evaluation stud* OR cross-over stud* OR latin square OR intervention stud* OR dietary intervention* |

**Supplemental Table 2. Excluded studies**

| **Excluded after full text review** | **reason for excluding** |
| --- | --- |
| Barnard ND, Cohen J, Jenkins DJ, Turner-McGrievy G, Gloede L, Jaster B et al. A low-fat vegan diet improves glycemic control and cardiovascular risk factors in a randomized clinical trial in individuals with type 2 diabetes. Diabetes care 2006; 29(8): 1777-1783. | - did not prescribe or assess red meat intake in the non-vegetarian diet |
| Belobrajdic DP, Frystyk J, Jeyaratnaganthan N, Espelund U, Flyvbjerg A, Clifton PM et al. Moderate energy restriction-induced weight loss affects circulating IGF levels independent of dietary composition. European journal of endocrinology 2010; 162(6): 1075-1082. | - weight loss diet |
| Gao J, Guo X, Wei W, Li R, Hu K, Liu X, Jiang W, Liu S, Wang W, Sun H, et al. The association of fried meat consumption with the gut microbiota and fecal metabolites and its impact on glucose homoeostasis, intestinal endotoxin levels, and systemic inflammation: A randomized controlled-feeding trial. Diabetes Care 2021; 44: 1970-9. | - did not include red meat in the fried meat intervention (only chicken and fish) |
| Gannon MC, Nuttall FQ, Neil BJ, Westphal SA. The insulin and glucose responses to meals of glucose plus various proteins in type II diabetic subjects. Metabolism 1988; 37(11): 1081-1088. | - control diet was 50 g glucose – not an actual “diet” but an artificial condition |
| González-Salazar LE, Pichardo-Ontiveros E, Palacios-González B, Vigil-Martínez A, Granados-Portillo O, Guizar-Heredia R, Flores-López A, Medina-Vera I, Heredia GCPK, Hernández-Gómez KG, et al. Effect of the intake of dietary protein on insulin resistance in subjects with obesity: a randomized controlled clinical trial. Eur J Nutr 2021; 60: 2435-47. | - weight loss diet |
| Hodgson JM, Ward NC, Burke V, Beilin LJ, Puddey IB. Increased Lean Red Meat Intake Does Not Elevate Markers of Oxidative Stress and Inflammation in Humans. The Journal of Nutrition 2007; 137(2): 363-367. | - same trial as Hodgson, Am J Clin Nutr, 2006 |
| Kahleova H, Matoulek M, Malinska H, Oliyarnik O, Kazdova L, Neskudla T et al. Vegetarian diet improves insulin resistance and oxidative stress markers more than conventional diet in subjects with Type 2 diabetes. Diabetic Medicine 2011; 28(5): 549-559. | - did not prescribe or assess red meat intake in the non-vegetarian diet |
| Khakimov B, Poulsen SK, Savorani F, Acar E, Gurdeniz G, Larsen TM et al. New Nordic Diet versus Average Danish Diet: A Randomized Controlled Trial Revealed Healthy Long-Term Effects of the New Nordic Diet by GC-MS Blood Plasma Metabolomics. J Proteome Res 2016; 15(6): 1939-1954. | - no outcomes of interest |
| Li J, Armstrong CL, Campbell WW. Effects of dietary protein source and quantity during weight loss on appetite, energy expenditure, and cardio-metabolic responses. Nutrients 2016; 8(2): 63. | - weight loss diet |
| Liao F-H, Shieh M-J, Yang S-C, Lin S-H, Chien Y-W. Effectiveness of a soy-based compared with a traditional low-calorie diet on weight loss and lipid levels in overweight adults. Nutrition 2007; 23(7-8): 551-556. | - weight loss diet |
| Mahon AK, Flynn MG, Stewart LK, McFarlin BK, Iglay HB, Mattes RD et al. Protein Intake during Energy Restriction: Effects on Body Composition and Markers of Metabolic and Cardiovascular Health in Postmenopausal Women. Journal of the American College of Nutrition 2007; 26(2): 182-189. | - weight loss diet |
| Mateo-Gallego R, Perez-Calahorra S, Cenarro A, Bea A, Andres E, Horno J et al. Effect of lean red meat from lamb v. lean white meat from chicken on the serum lipid profile: a randomised, cross-over study in women. The British journal of nutrition 2011; **107:** 1403-1407 | - no outcome data reported on glucose measures |
| Ouellet Vr, Weisnagel SJ, Marois J, Bergeron J, Julien P, Gougeon Rj et al. Dietary Cod Protein Reduces Plasma C-Reactive Protein in Insulin-Resistant Men and Women. The Journal of Nutrition 2008; 138(12): 2386-2391. | - Same trial as Ouellet, Diabetes Care, 2007 |
| Pearce KL, Clifton PM, Noakes M. Egg consumption as part of an energy-restricted high-protein diet improves blood lipid and blood glucose profiles in individuals with type 2 diabetes. Br J Nutr 2011; 105(4): 584-592 | - weight loss diet |
| Porter Starr KN, Connelly MA, Orenduff MC, McDonald SR, Sloane R, Huffman KM et al. Impact on cardiometabolic risk of a weight loss intervention with higher protein from lean red meat: Combined results of 2 randomized controlled trials in obese middle-aged and older adults. J Clin Lipidol 2019; 13(6): 920-931 | - weight loss diet |
| Sayer RD, Speaker KJ, Pan Z, Peters JC, Wyatt HR, Hill JO. Equivalent reductions in body weight during the Beef WISE Study: beef's role in weight improvement, satisfaction and energy. Obesity Science & Practice 2017; 3(3): 298-310. | - weight loss diet |
| Schmedes M, Balderas C, Aadland EK, Jacques H, Lavigne C, Graff IE et al. The Effect of Lean-Seafood and Non-Seafood Diets on Fasting and Postprandial Serum Metabolites and Lipid Species: Results from a Randomized Crossover Intervention Study in Healthy Adults. Nutrients 2018; 10(5): 598. | - TMAO measures not in usable form - no outcomes of interest |
| Schmedes M, Brejnrod AD, Aadland EK, Kiilerich P, Kristiansen K, Jacques H et al. The Effect of Lean-Seafood and Non-Seafood Diets on Fecal Metabolites and Gut Microbiome: Results from a Randomized Crossover Intervention Study. Mol Nutr Food Res 2019; 63(1): e1700976. | - no outcomes of interest |
| Schüler R, Markova M, Osterhoff MA, Arafat A, Pivovarova O, Machann J, Hierholzer J, Hornemann S, Rohn S, Pfeiffer AFH. Similar dietary regulation of IGF-1- and IGF-binding proteins by animal and plant protein in subjects with type 2 diabetes Eur J Nutr 2021; 60: 3499-504. | - duplicate data from Sucher, 2017 |
| Stella C, Beckwith-Hall B, Cloarec O, Holmes E, Lindon JC, Powell J et al. Susceptibility of human metabolic phenotypes to dietary modulation. J Proteome Res 2006; 5(10): 2780-2788. | - did not measure quantities of metabolites but presence only |
| Turner KM, Keogh JB, Meikle PJ, Clifton PM. Changes in lipids and inflammatory markers after consuming diets high in red meat or dairy for four weeks. Nutrients 2017; 9(8): 886. | - same trial as Turner, Am J Clin Nutr, 2015 |
| Wang Z, Bergeron N, Levison BS, Li XS, Chiu S, Jia X et al. Impact of chronic dietary red meat, white meat, or non-meat protein on trimethylamine N-oxide metabolism and renal excretion in healthy men and women. Eur Heart J 2019; 40(7): 583-594. | - multicomponent: red meat, white meat, non-animal protein in context of high sat fat or low sat fat diet |
| Willmann C, Heni M, Linder K, Wagner R, Stefan N, Machann J et al. Potential effects of reduced red meat compared with increased fiber intake on glucose metabolism and liver fat content: a randomized and controlled dietary intervention study. Am J Clin Nutr 2019; 109(2): 288-296. | - weight loss diet |

| **Excluded from meta-analysis** | reason for excluding |
| --- | --- |
| Belinova L, Kahleova H, Malinska H, Topolcan O, Vrzalova J, Oliyarnyk O, et al. Differential acute postprandial effects of processed meat and isocaloric vegan meals on the gastrointestinal hormone response in subjects suffering from type 2 diabetes and healthy controls: a randomized crossover study. PLoS One 2014; 9 (9): e107561 | - data in an unusable form |
| Cho CE, Taesuwan S, Malysheva OV, Bender E, Tulchinsky NF, Yan J, et al. Trimethylamine-N-oxide (TMAO) response to animal source foods varies among healthy young men and is influenced by their gut microbiota composition: A randomized controlled trial. Mol Nutr Food Res 2017; 61: 1600324. | - too few comparisons of TMAO for a meta-analysis (<3) |
| Markova M, Pivovarova O, Hornemann S, Sucher S, Frahnow T, Wegner K, Machann J, Petzke KJ, Hierholzer J, Lichtinghagen R, et al. Isocaloric Diets High in Animal or Plant Protein Reduce Liver Fat and Inflammation in Individuals With Type 2 Diabetes. Gastroenterology 2017; 152: 571-85. | - data in an unusable form – only correlation of insulin sensitivity with other outcomes |
| Samman S, Crossett B, Somers M, Bell KJ, Lai NT, Sullivan DR, Petocz P. Metabolic profiling of plasma amino acids shows that histidine increases following the consumption of pork. Diabetes Metab Syndr Obes 2014; 7: 203-10. | - data in an unusable form |
| Schmedes M, Aadland EK, Sundekilde UK, Jacques H, Lavigne C, Graff IE, et al. Lean-seafood intake decreases urinary markers of mitochondrial lipid and energy metabolism in healthy subjects: Metabolomics results from a randomized crossover intervention study. Mol Nutr Food Res 2016 Jul; 60: 1661-1672. | - too few comparisons of TMAO for a meta-analysis (<3) |
| Soucy J, Leblanc J. Protein meals and postprandial thermogenesis. Physiol Behav. 1999; 65: 705-709. | - data in an unusable form |

**Supplemental Table 3. Summary of studies included in meta-analysis**

| **Study** | **Population** | **Health status** | **Study design** | **Red meat exposure** | **Comparator diet exposure** | **Outcomes measured** |
| --- | --- | --- | --- | --- | --- | --- |
| Coelho et al 2021 | n = 10  40% male  24 ± 1 y  BMI: 23 ± 1 | healthy | Parallel  1 wk | Animal-based protein (beef and ham) | Plant-based protein (mycoprotein) | Fasting glucose  Fasting insulin  Insulin sensitivity |
| Crimarco et al 2020 | n = 36  30% male  50.2 ± 13.8 y  BMI: 27.9 ± 5.2 | healthy | Crossover  8 wk | Animal-based protein (mostly beef and pork with some chicken) | Plant-based protein  (no beef or pork) | Fasting glucose  Fasting insulin |
| Maki et al 2020 | n = 33  21% male  44.4 ± 2.4 y  BMI: 31.3 ± 0.8 | overweight, metabolic syndrome OR prediabetes | Crossover  4 wk | USDA lean beef diet (150 g beef replacing CHO in control diet) | USDA control diet  (less red meat <40g/d) | Fasting glucose  Fasting insulin  Insulin Sensitivity  Beta-cell function |
| Mitchell et al 2019 | RDA for pro  n = 15  100% male  74.7 ± 3.9 y  BMI 28.4 ± 5.1  2X RDA for pro  n = 14  100% male  73.7 ± 3.3  BMI: 28.2 ± 3.3 | healthy  >70 y | Parallel  10 wk | Twice the RDA for protein (some protein from red meat = approx. 1.1 svg/d beef, lamb, venison, or pork) | RDA for protein  (approx.. 0.2 svg/d beef, lamb, venison or pork) | Fasting glucose  Fasting insulin  Insulin sensitivity |
| Klementova et al 2019 | Healthy  n= 20  100% male  42.7 ± 7.1 y  BMI: 23.8 ± 1.5  Obese  n = 20  100% male  43 ± 7 y  BMI: 32.7 ± 3.9  T2D (same as Kahleova 2019)  n = 20  100% male  47.8 ± 8.2 y  BMI: 34.5 ±3.4 | healthy  obese  type 2 diabetes  (analyzed separately) | Crossover  Acute (< 24 h) | Pork patty | Plant-based “meat” patty | GLP-1 |
| Kahleova et al. 2019 | n=20  100% male  47.8 ± 8.2y  BMI 34.5 ± 3.4 | overweight or obese and type 2 diabetes | Crossover  Acute (< 24 h) | Pork patty | Plant-based “meat” patty | Postprandial glucose  Postprandial insulin  Insulin sensitivity  GLP-1 (also reported in Klementova, 2019)  GIP, Amylin |
| Hassanzadeh-Rostami et al. 2019 | Soy  n= 21  33% male  57.1 ± 7.3 y  BMI: 25.7 ±3.9  Legume  n = 20  35% male  59.6 ± 6 y  BMI: 27.3 ± 3.4  Red meat  n = 23  21% male  56.1 ± 7.2 y  BMI: 26.5 ± 3.2 | type 2 diabetes  age 40 – 65 | Parallel  8 wk | Red meat (type unspecified) 3 d/wk | Soy 3d/wk  Legumes 3d/wk | Fasting glucose  Fasting insulin  Insulin sensitivity  HbA1c |
| Palacios et al 2018 | n = 21  38% male  44.4 ±3.1 y  BMI: 30.4 ± 0.9 | overweight or obese | Crossover  2 wk | Lean pork breakfast | Refined CHO breakfast | Fasting glucose  Fasting insulin  Postprandial glucose  Postprandial insulin  Insulin sensitivity  Beta-cell function |
| O’Connor et al 2018 | n = 41  32% male  46 ± 2 y  BMI: 30.5 ± 0.6 | overweight or obese | Crossover  5 wk | Mediterranean diet with 500 g/wk lean red meat (beef or pork tenderloin) | Mediterranean diet with 200 g/wk lean red meat (beef or pork tenderloin) | Fasting glucose  Fasting insulin  Insulin sensitivity |
| Sucher et al 2017 | Plant pro  n = 19  63% male  63.7 ± 1.5 y  BMI: 29.4 ± 1  Animal pro  n = 18  67% male  65 ± 1.4 y  BMI: 31 ± 0.8 | type 2 diabetes and HbA1c >6% | Parallel  6 wk | Animal protein  Type of red meat unspecified, but sample menu included ham | Plant protein (pea protein) | Fasting glucose  Fasting insulin  Insulin sensitivity  HbA1c  Plasma FFA |
| Turner et al 2016 | n = 43  44% male  50.8 ± 16 y  BMI: 30 ± 3.5 | overweight or obese | Crossover  Acute (24 h) | Lean beef | Low fat dairy | Postprandial glucose  Postprandial insulin |
| Aadland et al 2016 | n = 19-20  35% male  50.6 ± 3.4 y  BMI: 25.6 ± 0.7 | healthy | Crossover  4 wk | Non-seafood protein (lean beef or pork, poultry, egg, dairy) | Lean seafood | Fasting glucose  Fasting insulin  Insulin sensitivity  Plasma FFA  Adiponectin  Glucagon |
| Douglas et al 2015 | n = 24  Sex not reported  23 ± 1 y  BMI: 23.4 ± 0.6 | healthy, normal to overweight | Crossover  Acute (< 24 h) | 96% lean ground beef | soy protein concentrate | GLP-1 |
| Turner et al 2015 | n = 47  38% male  47.8 ± 13 y  BMI: 31.1 ± 5.1 | overweight or obese | Crossover  4 wk | >200 g/d lean, unprocessed red meat (type unspecified) | >200 g/d chicken or fish  4 – 6 svg/d low fat dairy | Fasting glucose  Fasting insulin  OGTT  Insulin sensitivity |
| Hill et al 2015 | DASH plant pro  n = 21  43% male  45.3 ± 6.7 y  BMI: 34.7 ± 3.6  DASH-BOLD  n = 20  45% male  46.2 ± 9.4 y  BMI: 34.6 ± 3.7  DASH-BOLD+  n = 21  48% male  46.4 ± 8.5 y  BMI: 35.1 ± 4.5 | overweight or obese and metabolic syndrome | Parallel  5 wk | DASH-BOLD (139 g/d lean beef)  DASH-BOLD+ (196 g/d lean beef) | DASH-plant (12 g/d lean beef) | Fasting insulin  Fasting glucose |
| van Nielen et al 2014 | n = 15  0% male  61 ± 5 y  BMI: NR | postmenopausal women with abdominal obesity | Crossover  4 wk | High protein mixed meats (mostly pork and chicken) | High protein soy based | Fasting glucose  Fasting insulin  Insulin sensitivity |
| Roussell et al 2012 | n = 36  42% male  50 ± 1.4 y  BMI: 25.7 ± 0.5 | elevated LDL-c | Crossover  5 wk | DASH-BOLD (113 g/d lean beef)  DASH-BOLD+ (153 g/d lean beef) | HAD (20 g/d lean beef)  DASH (28 g/d lean beef) | Fasting glucose  Fasting insulin |
| Navas-Carretero et al 2009 | n = 25  0% male  Age range 18 -30  BMI: 22.1 ± 2.2 | premenopausal women, low iron stores | Crossover  8 wk | Red meat (4 or 5 portions/wk, type unspecified) | Oily fish (e.g., salmon, tuna, sardines) (4 or 5 portions/wk) | Fasting glucose  Fasting insulin  Insulin sensitivity |
| Azadbakht et al 2007 | n = 42  0% male  Age NR  BMI NR | postmenopausal women with metabolic syndrome | Crossover  8 wk | DASH – red meat (type unspecified) | DASH soy protein  DASH soy nut (roasted soybeans) | Fasting glucose  Fasting insulin  Insulin sensitivity |
| Ouellet et al 2007 | n = 19  53% male  53.8 ± 2.6 y (male)  55.4 ± 2.9 (female) | overweight/obese  insulin resistant | Crossover  4 wk | Lean beef, pork, veal | Cod fillets | Insulin sensitivity  Beta-cell function |
| Hodgson et al 2006 | Usual diet  n = 31  58% male  60 ± 9.9 y  BMI: 27.9 ± 4  Red meat  n = 29  69% male  57.2 ± 7.3 y  BMI: 27.5 ± 3.1 | hypertension | Parallel  8 wk | Lean red meat (replace CHO, type unspecified) | Usual diet | Fasting insulin  Fasting glucose  Insulin sensitivity  HbA1c |

Abbreviations: BOLD = Beef in an Optimal Lean Diet, CHO = carbohydrate, DASH = Dietary Approaches to Stop Hypertension, FFA = free fatty acids, GIP = gastric inhibitory peptide, GLP-1 = glucagon like peptide – 1, HAD = Healthy American Diet, HbA1c = glycated hemoglobin, HOMA-IR = homeostatic model assessment of insulin resistance, LDL-c = low density lipoprotein cholesterol, OGTT = oral glucose tolerance test

**Supplemental Table 4.** One-study-removed at a time sensitivity analysis for the effect of red meat intake, compared to less or no red meat, on markers of glycemia and insulinemia.

| **Insulin Sensitivity** |  |  |  |
| --- | --- | --- | --- |
| **Reference** | **Effect Estimate SMD^1^** | **95% CI** | **P-value^1^** |
| Coelho, 2021 | -0.13 | -0.42, 0.16 | 0.372 |
| Maki, et al., 2020 | -0.11 | -0.42, 0.20 | 0.483 |
| Hassanzadeh-Rostami, et al., 2019 | -0.14 | -0.43, 0.16 | 0.361 |
| Kahleova, et al., 2019 | -0.14 | -0.44, 0.15 | 0.347 |
| Mitchell, et al., 2019 | -0.14 | -0.43, 0.15 | 0.330 |
| O’Connor, et al. 2018 | -0.13 | -0.44, 0.15 | 0.410 |
| Palacios, et al., 2018 | -0.10 | -0.40, 0.20 | 0.507 |
| Sucher, et al., 2017 | -0.15 | -0.44, 0.14 | 0.307 |
| Aadland, et al., 2016 | -0.10 | -0.39, 0.20 | 0.517 |
| Turner, et al., 2015 | -0.13 | -0.45, 0.18 | 0.399 |
| Van Nielen, et al. 2014 | -0.16 | -0.45, 0.13 | 0.273 |
| Navas-Carretero, et al., 2009 | -0.12 | -0.42, 0.18 | 0.430 |
| Azadbakht, et al., 2007 | 0.01 | -0.14, 0.17 | 0.879 |
| Ouellet, 2007 | -0.08 | -0.37, 0.21 | 0.598 |
| Hodgson, et al., 2006 | -0.10 | -0.40, 0.20 | 0.507 |
| **HOMA-IR** |  |  |  |
| **Reference** | **Effect Estimate SMD^1^** | **95% CI** | **P-value^1^** |
| Coelho, 2021 | 0.11 | -0.25, 0.48 | 0.543 |
| Kahleova, et al., 2019 | 0.12 | -0.27, 0.50 | 0.553 |
| Mitchell, et al., 2019 | 0.15 | -0.21, 0.51 | 0.411 |
| O’Connor, et al. 2018 | 0.13 | -0.27, 0.52 | 0.527 |
| Sucher, et al., 2017 | 0.15 | -0.21, 0.52 | 0.408 |
| Aadland, et al., 2016 | 0.09 | -0.29, 0.47 | 0.641 |
| Turner, et al., 2015 | 0.14 | -0.26, 0.53 | 0.500 |
| Van Nielen, et al. 2014 | 0.14 | -0.24, 0.51 | 0.472 |
| Navas-Carretero, et al., 2009 | 0.10 | -0.29, 0.49 | 0.603 |
| Azadbakht, et al., 2007 | -0.06 | -0.20, 0.07 | 0.381 |
| Hodgson, et al., 2006 | 0.09 | -0.29, 0.46 | 0.648 |
| **Fasting Glucose** |  |  |  |
| **Reference** | **Effect Estimate SMD^1^** | **95% CI** | **P-value^1^** |
| Coelho, et al., 2021 | 0.10 | -0.06, 0.27 | 0.205 |
| Crimarco, et al., 2020 | 0.14 | -0.03, 0.32 | 0.113 |
| Maki, et al., 2020 | 0.10 | -0.07, 0.28 | 0.245 |
| Mitchell, et al., 2019 | 0.14 | -0.03, 0.31 | 0.110 |
| Hassanzadeh-Rostami, et al., 2019 | 0.15 | -0.01, 0.32 | 0.065 |
| Palacios, et al., 2018 | 0.10 | -0.07, 0.27 | 0.238 |
| O’Connor, et al. 2018 | 0.15 | -0.02, 0.32 | 0.078 |
| Sucher, et al., 2017 | 0.15 | -0.01, 0.32 | 0.063 |
| Aadland, et al., 2016 | 0.12 | -0.05, 0.30 | 0.168 |
| Turner, et al., 2015 | 0.13 | -0.06, 0.31 | 0.171 |
| Hill, et al., 2015 | 0.12 | -0.05, 0.29 | 0.173 |
| Van Nielen, et al. 2014 | 0.14 | -0.03, 0.32 | 0.098 |
| Roussell, et al., 2012 | 0.12 | -0.06, 0.30 | 0.195 |
| Navas-Carretero, et al., 2009 | 0.14 | -0.04, 0.31 | 0.126 |
| Azadbakht, et al., 2007 | 0.08 | -0.07, 0.23 | 0.300 |
| Hodgson, et al., 2006 | 0.11 | -0.07, 0.28 | 0.226 |
| **Fasting Insulin** |  |  |  |
| **Reference** | **Effect Estimate SMD^1^** | **95% CI** | **P-value^1^** |
| Coelho, 2021 | 0.09 | -0.16, 0.33 | 0.491 |
| Crimarco, et al., 2020 | 0.09 | -0.17, 0.35 | 0.513 |
| Maki, et al., 2020 | 0.07 | -0.19, 0.33 | 0.587 |
| Mitchell, et al., 2019 | 0.10 | -0.15, 0.35 | 0.433 |
| Hassanzadeh-Rostami, et al., 2019 | 0.09 | -0.16, 0.34 | 0.487 |
| Palacios, et al., 2018 | 0.07 | -0.19, 0.33 | 0.595 |
| O’Connor, et al. 2018 | 0.09 | -0.17, 0.35 | 0.505 |
| Sucher, et al., 2017 | 0.10 | -0.15, 0.35 | 0.431 |
| Aadland, et al., 2016 | 0.074 | -0.19, 0.32 | 0.616 |
| Turner, et al., 2015 | 0.10 | -0.16, 0.36 | 0.448 |
| Hill, et al., 2015 | 0.10 | -0.15, 0.35 | 0.452 |
| Van Nielen, et al. 2014 | 0.10 | -0.15, 0.35 | 0.415 |
| Roussell, et al., 2012 | 0.06 | -0.20, 0.32 | 0.655 |
| Navas-Carretero, et al., 2009 | 0.07 | -0.19, 0.33 | 0.585 |
| Azadbakht, et al., 2007 | -0.02 | -0.12, 0.09 | 0.764 |
| Hodgson, et al., 2006 | 0.07 | -0.18, 0.32 | 0.533 |
| **Postprandial Glucose** |  |  |  |
| **Reference** | **Effect Estimate SMD^1^** | **95% CI** | **P-value^1^** |
| Kahleova, et al., 2019 | -0.49 | -0.79, -0.19 | **0.001** |
| Palacios, et al., 2018 | -0.36 | -0.62, -0.11 | **0.005** |
| Turner, et al., 2016 | -0.51 | -0.88, -0.13 | **0.009** |
| **Postprandial Insulin** |  |  |  |
| **Reference** | **Effect Estimate SMD^1^** | **95% CI** | **P-value^1^** |
| Kahleova, et al., 2019 | -0.27 | -0.74, 0.21 | 0.272 |
| Palacios, et al., 2018 | -0.81 | -2.36, 0.73 | 0.302 |
| Turner, et al., 2016 | -1.06 | -2.13, 0.01 | 0.051 |
| **HbA1c** |  |  |  |
| **Reference** | **Effect Estimate SMD^1^** | **95% CI** | **P-value^1^** |
| Hassanzadeh-Rostami, et al., 2019 | 0.35 | -0.05, 0.75 | 0.089 |
| Sucher, et al., 2017 | 0.07 | -0.71, 0.85 | 0.859 |
| Hodgson, et al., 2006 | -0.11 | -0.37, 0.58 | 0.668 |
| **Pancreatic beta-cell function** |  |  |  |
| **Reference** | **Effect Estimate SMD^1^** | **95% CI** | **P-value^1^** |
| Maki, et al., 2020 | -0.06 | -0.40, 0.28 | 0.743 |
| Palacios, et al., 2018 | -0.23 | -0.51, 0.05 | 0.106 |
| Ouellet, et al, 2007 | -0.09 | -0.40, 0.22 | 0.581 |
|  |  |  |  |
|  |  |  |  |
| **GLP-1** |  |  |  |
| **Reference** | **Effect Estimate SMD^1^** | **95% CI** | **P-value^1^** |
| Klementova, et al., 2019 (healthy) | -0.58 | -1.47, 0.32 | 0.206 |
| Klementova, et al., 2019 (obese) | -0.62 | -1.56, 0.31 | 0.192 |
| Klementova, et al., 2019 (T2D) | -0.41 | -1.10, 0.27 | 0.235 |
| Douglas, et al., 2015 | -0.91 | -1.27, -0.55 | **<0.001** |

^1^effect estimates and p-values from random effects models

**Supplemental Table 5**. Risk of bias assessment

| **Author** | **Outcome** | **Overall Bias** |
| --- | --- | --- |
| Coelho 2021 | Insulin/Glucose measures | Some concerns |
| Maki 2020 | Insulin/Glucose measures | Low |
| Crimarco 2020 | Insulin/Glucose measures | High |
| Mitchell 2019 | Insulin/Glucose measures | Some concerns |
| Kahleova 2019 | Insulin/Glucose measures | Low |
| Klementova 2019 | GLP-1 | Low |
| Hassanzadeh-Rostami 2019 | Insulin/Glucose measures | Some concerns |
| Palacios 2018 | Insulin/Glucose measures | Low |
| O'Connor 2018 | Insulin/Glucose measures | Low |
| Sucher 2017 | Insulin/Glucose measures | Some concerns |
| Douglas 2015 | GLP-1 | Low |
| Turner 2015 | Insulin/Glucose measures | Low |
| Aadland 2016 | Insulin/Glucose measures | Low |
| Turner 2016 | Insulin/Glucose measures | Low |
| Hill 2015 | Insulin/Glucose measures | Low |
| van Nielen 2014 | Insulin/Glucose measures | Low |
| Roussell 2011 | Insulin/Glucose measures | Low |
| Navas-Carretero 2009 | Insulin/Glucose measures | Some concerns |
| Azadbakht 2007 | Insulin/Glucose measures | Low |
| Ouellet 2007 | Insulin/Glucose measures | Low |
| Hodgson 2006 | Insulin/Glucose measures | Low |

**Supplemental Table 6.** Analysis of publication bias for studies evaluating the effect of red meat intake, compared to less or no red meat, on markers of glycemia and insulinemia.

| **Outcome** | **P-value^1^** |
| --- | --- |
| Insulin sensitivity | 0.608 |
| HOMA-IR | 0.819 |
| Fasting glucose | 0.889 |
| Fasting insulin | 0.966 |
| Postprandial glucose^2^ | 0.653 |
| Postprandial insulin^2^ | 0.096 |
| HbA1c^2^ | 0.824 |
| Pancreatic beta-cell function^2^ | 0.853 |
| GLP-1^2^ | 0.023 |

^1^Egger’s Regression

^2^Interpret with caution due to <10 studies

**Supplemental Figures**





**Supplemental Figure 1.** Forest plot of the meta-analysis on the effect of red meat on postprandial insulin response. Values are the standardized mean differences (SMD) for postprandial insulin area under the curve (AUC) between diets with red meat intake and diets with less or no red meat intake.





**Supplemental Figure 2.** Forest plot of the meta-analysis on the effect of red meat on HbA1c. Values are the standardized mean differences (SMD) for HbA1c between diets with red meat intake and diets with less or no red meat intake.

**
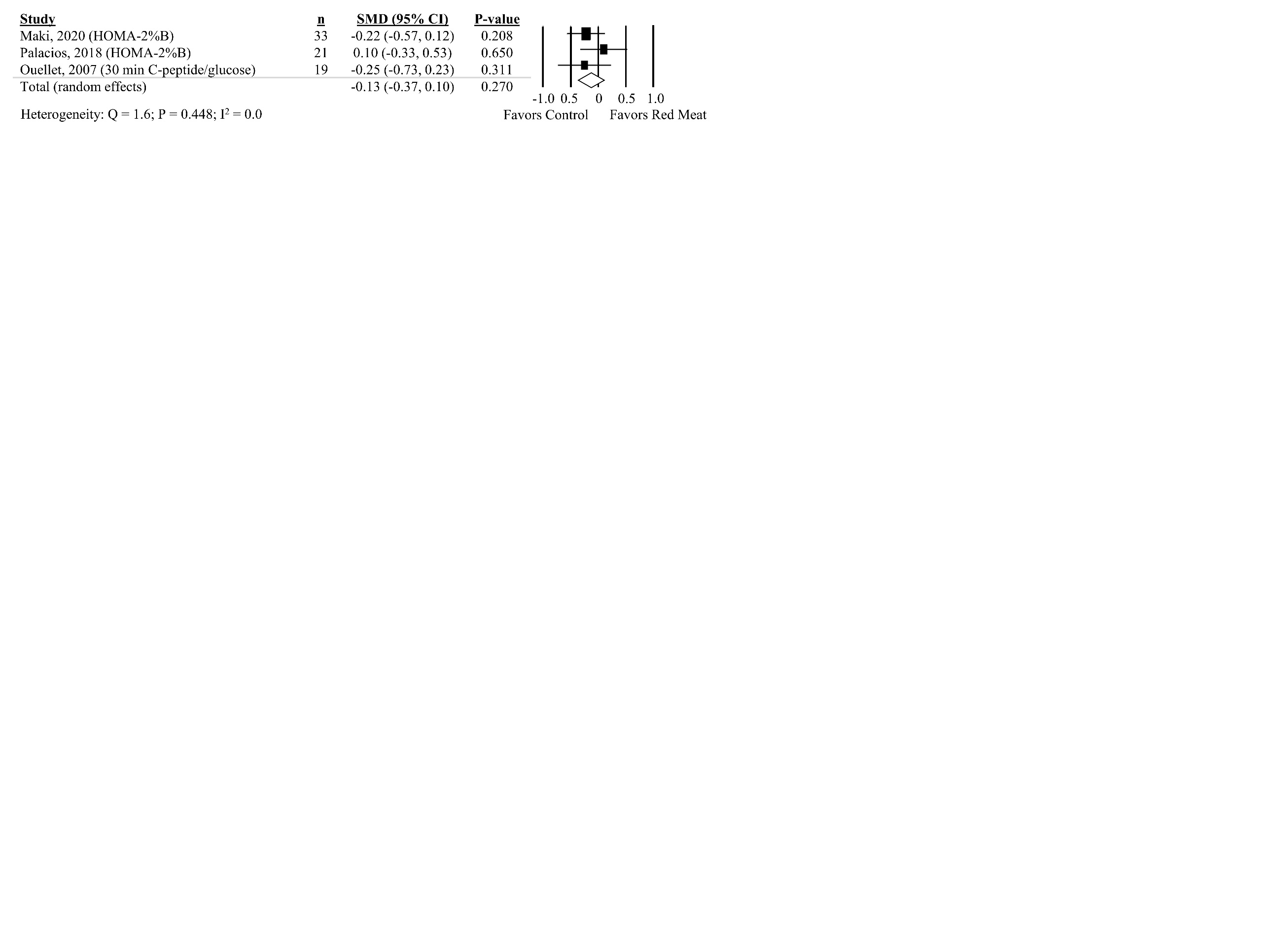
**

**Supplemental Figure 3.** Forest plot of the meta-analysis on the effect of red meat on pancreatic beta-cell function. Values are the standardized mean differences (SMD) for pancreatic beta-cell function between diets with red meat intake and diets with less or no red meat intake. Ouellet, 2007 also measured beta-cell function by Disposition Index (30 min C-peptide/glucose X insulin sensitivity). A separate analysis using Disposition Index instead of 30 min C-peptide/glucose provided similar results.


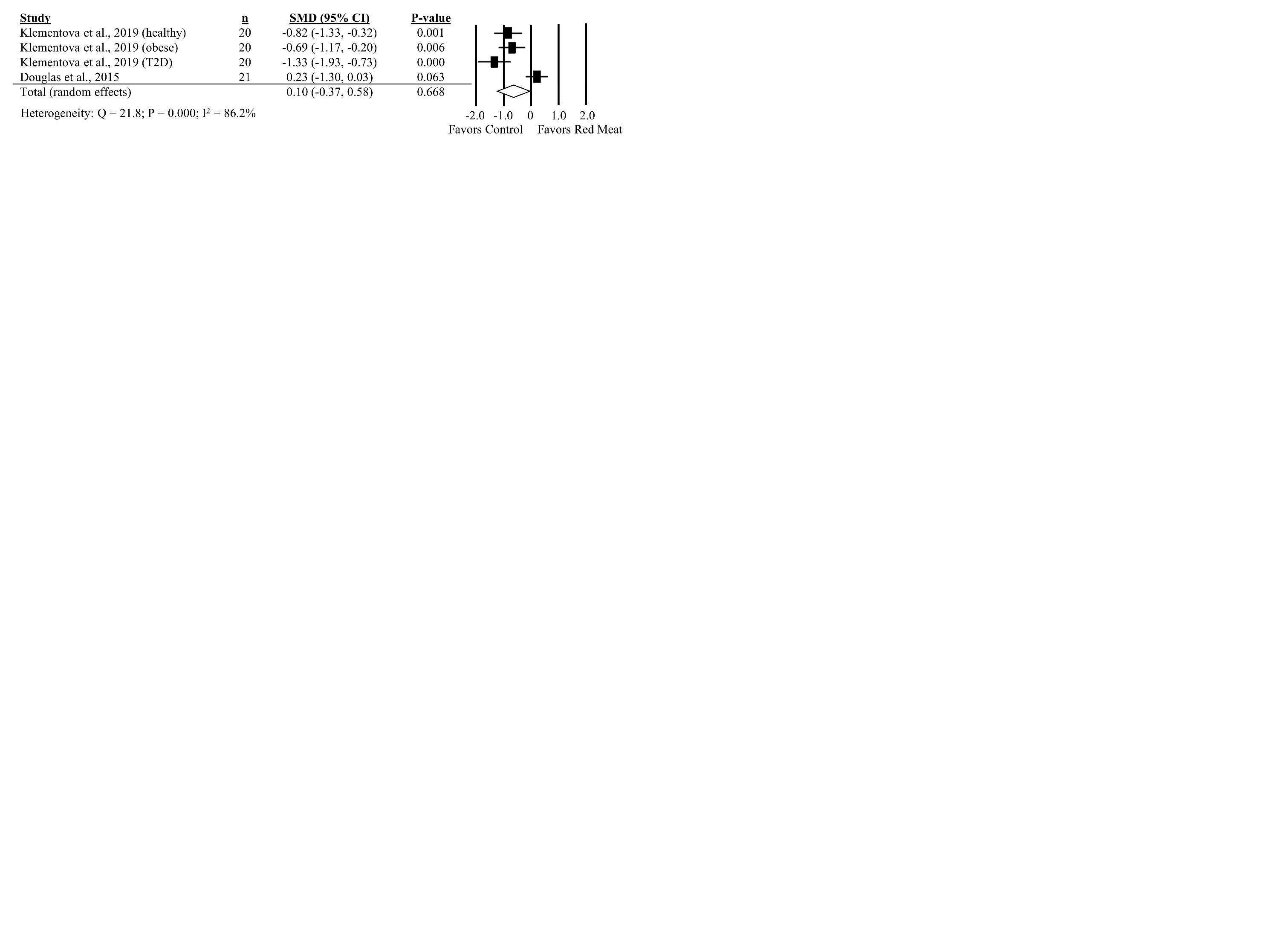


**Supplemental Figure 4.** Forest plot of the meta-analysis on the effect of red meat on GLP-1. Values are the standardized mean differences (SMD) for GLP-1 between diets with red meat intake and diets with less or no red meat intake.
